# Temporally and spatially dynamic germ cell niches in *Botryllus schlosseri* revealed by expression of a TGF-beta family ligand and *vasa*

**DOI:** 10.1186/s13227-016-0047-5

**Published:** 2016-04-11

**Authors:** Adam D. Langenbacher, Anthony W. De Tomaso

**Affiliations:** Department of Molecular, Cellular and Developmental Biology, University of California Santa Barbara, Santa Barbara, CA 93106-9610 USA; Department of MCD Biology, UCLA, Los Angeles, CA 90095 USA

**Keywords:** Germ cells, Follicle cells, Reproduction, Budding, Blastogenesis, Botryllus, Tunicate, Transforming growth factor beta

## Abstract

**Background:**

Germ cells are specified during early development and are responsible for generating gametes in the adult. After germ cells are specified, they typically migrate to a particular niche in the organism where they reside for the remainder of its lifetime. For some model organisms, the specification and migration of germ cells have been extensively studied, but how these events occur in animals that reproduce both sexually and asexually is not well understood.

**Results:**

We have identified a novel TGF-β family member in *Botryllus schlosseri*, *tgfβ*-*f*, and found that it is expressed by follicle cell progenitors and the differentiated follicle and support cells surrounding the maturing gametes. Using the expression of *tgfβ*-*f* and the germ cell marker *vasa*, we have found that nearly all germ cells in *Botryllus* are associated with *tgfβ*-*f*-expressing follicle progenitors in clusters consisting solely of those two cell types. These clusters were mostly small, consisting of ten or fewer cells, and generally contained between a 2:1 and 1:1 ratio of follicle progenitors to germ cells. Clusters of germ and follicle progenitor cells were primarily localized to niches in the primary and secondary buds, but could also be found in other locations including the vasculature. We analyzed the location of germ cell clusters throughout the asexual life cycle of *Botryllus* and found that at the stage when germ cells are first detected in the secondary bud niche, a dramatic change in the size and location of germ/follicle cell clusters also occurred.

**Conclusions:**

Our findings suggest that germ/follicle cell clusters have predictable migratory patterns during the weekly asexual developmental cycle in *Botryllus*. An increased number of small clusters and the presence of clusters in the vasculature coinciding with the appearance of clusters in the secondary bud suggest that fragmentation of clusters and the migration of smaller clusters through the vasculature may be an important aspect of *Botryllus* reproductive biology, ensuring the transmission of the germline to subsequent asexual generations.

**Electronic supplementary material:**

The online version of this article (doi:10.1186/s13227-016-0047-5) contains supplementary material, which is available to authorized users.

## Background

Germ cells are specified and segregate early during embryonic development in animals and persist through adulthood to generate the gametes needed to ensure that an organism’s genetic material is passed on to the next generation. Examination of a number of metazoan species has revealed that two different strategies are employed for specifying the germline, termed preformation and epigenesis [1–3]. In preformation, a cache of maternal determinants called the germ plasm is segregated into a particular area of the oocyte or embryo, promoting a germline fate in the cells that inherit it. In epigenesis, inductive signals result in the differentiation of germ cells from another tissue, usually at later stages. Interestingly, the tissue from which primordial germ cells are specified varies greatly among the metazoans that have been examined [1].

Regardless of their method of specification, primordial germ cells typically migrate to a specific niche in the developing embryo that will eventually support the production of gametes. Evidence from multiple species suggests that germ cells possess inherent motility, but require external factors to control this migration [4]. For example, the chemokine SDF1 in zebrafish and lipid molecules in *Drosophila* serve as directional cues to guide germ cells to the gonadal niche. While the precise mechanisms differ between organisms, G protein-coupled receptor and lipid signaling appear to play a conserved role in the process of directional germ cell migration [3–5].

*Botryllus schlosseri* is a colonial marine chordate in the subphylum Tunicata, a group of invertebrates thought to be the closest living relatives of the vertebrates [6]. Colonies of *Botryllus* are organized into star-shaped systems, with the filter-feeding adults (called zooids) occupying the center. When mature and under favorable environmental conditions, zooids produce gametes for sexual reproduction. Each week, all zooids in a colony undergo apoptosis and are replaced in a cyclical, asexual budding process called blastogenesis [7, 8]. Two generations of buds develop laterally to the zooids, with “primary buds” undergoing organogenesis growing from the zooids and newly formed “secondary buds” emerging from the epithelia of the primary buds [9]. Asexual development of a bud requires 2 weeks to complete, with 1 week spent as a secondary bud and the other spent as a primary bud, and the development of the buds in a colony is synchronized.

Thus each day, *Botryllus* colonies consist of three simultaneous generations of individuals at one of seven predictable, sequential stages defined based on developmental characteristics of the primary and secondary buds. These stages are termed A1, A2, B1, B2, C1, C2, and D [10]. Secondary buds begin development at stage A1 as evaginations of the epithelia of primary buds and protrude anteriorly at stage A2. At stage B1, secondary bud growth continues and a heartbeat is initiated in the primary buds. The secondary bud forms a double-vesicle structure with an independent inner epithelium at stage B2. This inner epithelium subsequently undergoes a process of invaginations to form the major organ rudiments during stages C1 and C2. Stage D, also termed “takeover,” is characterized by apoptosis of the adult zooids and their removal by phagocytic cells. Following takeover, the fully developed primary buds open their siphons and become feeding, adult zooids. The previous generation’s secondary buds then become primary buds, producing new budlets of their own. The development of gametes is also highly synchronized with the blastogenic cycle, with spermatogenesis completing in zooids at stage B1 and vitellogenesis of oocytes completing at stage A1 [8, 11–13].

Individuals in a *Botryllus* colony are interconnected by an extracorporeal vasculature that is capable of parabiosing with an adjacent individual, creating a hematopoietic chimera [14–17]. Intriguingly, fusion between two colonies sometimes results in the complete replacement of the germline of one colony by the other in a phenomenon known as germline parasitism [11, 18, 19]. Germline parasitism can also be replicated by manually transplanting a FACS-isolated population of cells high in aldehyde dehydrogenase activity, a biomarker for stem cells in vertebrates [20, 21], suggesting that mobile germline stem cells are present in the circulation of adult *Botryllus*.

Given its ability to reproduce both sexually and asexually, *Botryllus* is a powerful model for understanding how the specification, migration, and differentiation of the germline are controlled in animals that grow by asexual budding and for comparing these mechanisms to those employed during embryonic development. The germline is specified very early in *Botryllus* development and can be visualized as maternally deposited *vasa* mRNA segregating into a posterior lineage of cells during cleavage stages [22]. Exactly how or if this early lineage migrates to seed the gonadal niches of the oozooid following metamorphosis has not yet been clearly determined, and studies on a related species, *Botryllus primigenus*, indicate that in at least some botryllids, germ cells can arise in adults by epigenesis [23–27]. Expression of *vasa* appears to be a universal marker of the germline [28], and it expectedly marks germ cells and gonads in juvenile and adult *Botryllus*. Based on the expression of *vasa* and other genes, germ cells in *Botryllus* were found to sometimes appear in the vasculature, suggesting they may represent a mobile germline in an adult animal [22, 29, 30]. This mobility is a mechanism by which the germline can escape the weekly turnover of bodies that occurs during blastogenesis and explains the phenomenon of germline parasitism [31]. Our laboratory has recently shown that *Botryllus* germ cells express *sphingosine*-*1*-*phosphate receptor* and that inhibition of sphingosine-1-phosphate signaling prevents migration of germ cells to newly developing buds [30]. However, despite having a number of markers for germ cells in *Botryllus*, the precise spatiotemporal dynamics of these cells with respect to the blastogenic cycle has not previously been ascertained.

Here, we report the identification of a *B. schlosseri* TGF-β superfamily member ligand homolog (Tgfβ-f) that is expressed by follicle cell progenitors and the follicle and support cells surrounding the developing gametes. We found that nearly all germ cells in *Botryllus* juveniles were associated with *tgfβ*-*f*-expressing follicle cell progenitors in clusters consisting of solely these two cell types. We used double fluorescent in situ hybridization to examine both the temporal and spatial localization of germ/follicle cell clusters (GFCs) in *Botryllus*. Our findings indicate that the localization of GFCs is highly dynamic with respect to the asexual life cycle of *Botryllus* and suggest that these GFCs may be migratory during a particular phase of blastogenesis.

## Methods

### Animals

*Botryllus* colonies were raised on glass slides in a mariculture system with circulating 0.5 µm filtered seawater at 18–20 °C. Animals were fed daily with live algae. Developmental staging of animals was performed by examination of primary and secondary buds under a dissecting microscope [10]. All individuals used in this study were siblings from an inbred line maintained in our laboratory (SB962).

### Cloning of genes used for in situ hybridization analysis

Total RNA was isolated from *Botryllus* colonies with the NucleoSpin RNA II kit (MN, 740955), and SuperScript II Reverse Transcriptase (Life Technologies, 18064-014) was used to synthesize cDNA primed by random primers (Life Technologies, 48190-011). PCR was performed with Advantage cDNA Polymerase (Clontech, 639105), and products were cloned into the pGEM-T Easy vector (Promega, A1360).

### Synthesis of antisense RNA probes

Constructs were linearized, and approximately 1 µg of template was used in an in vitro transcription reaction with SP6 or T7 RNA polymerase (Roche, 10810274001, 10881767001). For digoxigenin labeling of probes, DIG RNA labeling mix (Roche, 11277073910) was added to the reaction. Dinitrophenol labeling was accomplished by adding dinitrophenol-11-UTP (PerkinElmer, NEL555001EA), UTP, CTP, GTP, and ATP (Roche, 11277057001) to the reaction at final concentrations of 0.35, 0.65, 1, 1, and 1 mM, respectively. RNases were inhibited with Protector RNase Inhibitor (Roche, 03335399001). The transcription reaction was incubated at 37 °C for 2 h, and then RNase-free DNase I (Roche, 04716728001) was added to remove the plasmid template. Probes were then precipitated twice with LiCl/ethanol to maximize removal of unincorporated labeled nucleotides [32].

Antisense probes used in this study include *tgfβ*-*f* and *vasa*. Primer sequences (5′–3′) used for amplifying these genes include:

- vasa-F: AGGCACTATGATTCAGCCTGTG
- vasa-R: ATCATAATCACCCGTCTCGCG
- tgfβ-f-F: CATGGATTCTTGCAGGAGAG
- tgfβ-f-R: GTTACCGAACTTTCTGACCC.

### Fluorescent whole-mount in situ hybridization

Fluorescent whole-mount in situ hybridization was performed as described previously [33]. Imaging of labeled samples was performed using an Olympus FLV1000S Spectral Laser Scanning Confocal.

### Cell measurements and cluster analysis

Confocal z-stacks were manually analyzed using the Fiji package for ImageJ [34, 35]. Three-dimensional reconstructions and projections of confocal z-stacks were made using either the ImageJ or Imaris software. A total of 2682 *vasa*-positive germ cells and 2936 *tgfβ*-*f*-positive follicle cells, comprising 241 GFCs from 21 individual colonies, were utilized for this analysis. Each cell was given an identifying number based on the GFC it belonged to and the stage of the animal it was from, and its association was recorded as follows: zooid, primary bud, primary bud niche, secondary bud, tunic, or vasculature. Since mRNA is localized to the cytoplasm, we used the extent of the staining by in situ probes as an estimate of the actual size of each cell. The diameter of each cell was estimated by manually selecting the z-stack image plane it appeared largest in and drawing a region of interest around the cell’s perimeter in ImageJ. Feret’s diameter was then calculated using the Measure function of ImageJ. Raw image data are available upon request.

### Phylogenetic analysis

Protein sequences used for phylogenetic analysis were downloaded from the NCBI database. A full list of the species and GenBank or NCBI Reference IDs for each sequence used is found in Additional file 1: Data S1. Full-length TGF-β protein sequences were aligned using the MUSCLE algorithm in the MEGA6 application [36], and gaps were trimmed using the *automated1* method in the trimAl software to remove potentially poorly aligned regions [37], producing a 236 position alignment. The trimmed protein alignments are provided in Additional file 2: Data S2. Phylogenetic analysis of TGF-β family members was performed with the software RAxML using a maximum likelihood method with 504 replicates for bootstrap, the JTT substitution matrix, and empirical frequencies [38]. Another phylogenetic analysis was performed using the software MrBayes with four independent runs of one million generations, with trees sampled every 100 generations, 25 % of sampled values discarded as burn-in, and the mixed model mode [39–41]. RAxML software and MrBayes software were accessed using the CIPRES Science Gateway [42].

## Results

### Identification of Tgfβ-f

**Fig. 1:**
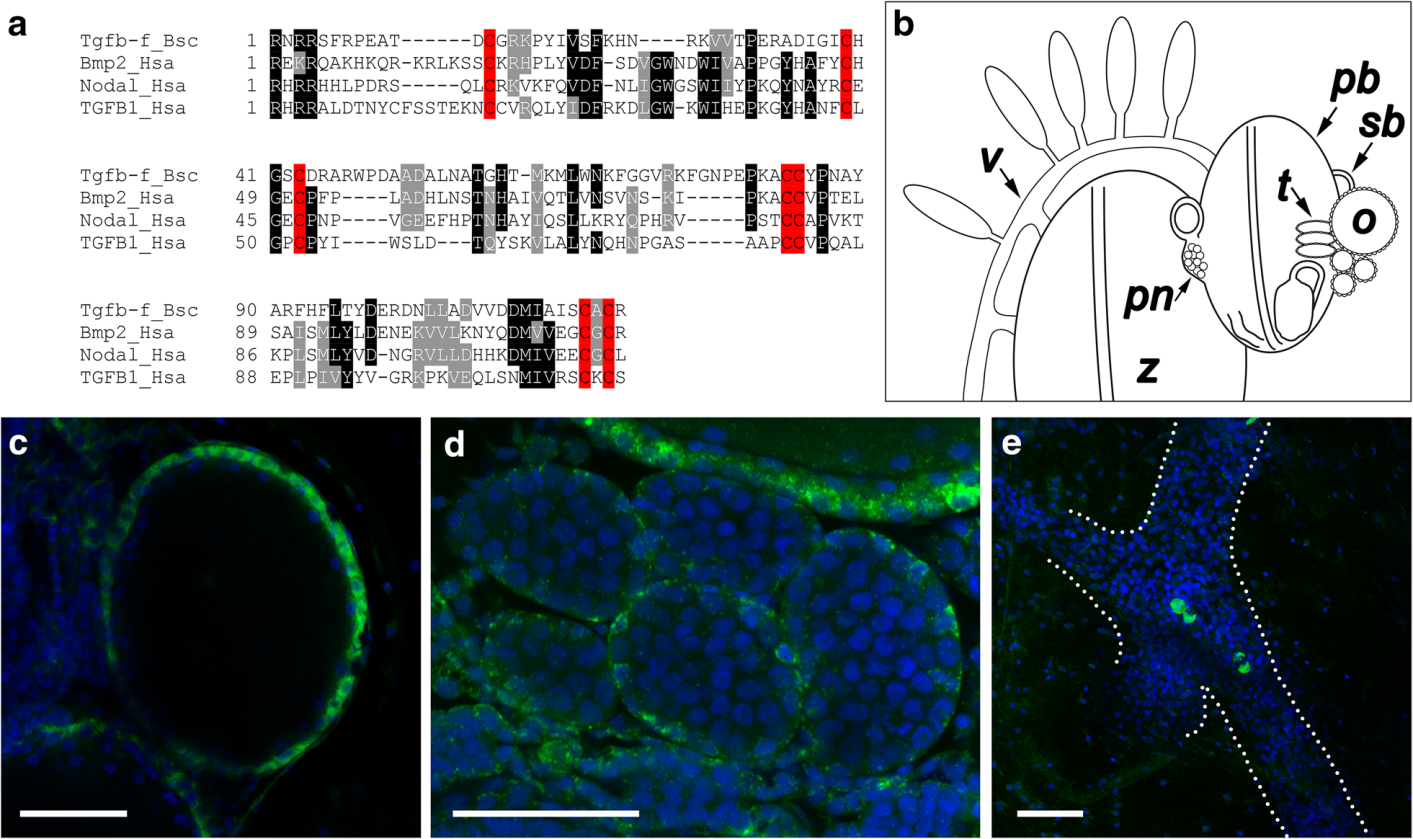
Tgfβ-f is a TGF-β family member expressed by follicle cells surrounding the developing gonads of *B. schlosseri*. **a** Alignment of the TGF-β domain of *Botryllus* (Bsc) Tgfβ-f with the TGF-β domains of human (Hsa) Bmp2, Nodal, and TGFβ1. Sites where amino acids are conserved in at least 75 % of the sequences are highlighted *black*, and sites with at least 75 % amino acid similarity are highlighted *gray*. Conserved cysteine residues are highlighted *red*. **b** Schematic of a *Botryllus* colony. The adult zooid (*z*) is connected to a developing primary bud (*pb*) that contains maturing gonads including oocytes (*o*) and testes (*t*). Secondary buds (*sb*) are present anterior to the maturing gonads and the primary bud niche (*pn*) that contains germ cells. The bodies and buds of a colony are interconnected by an extracorporeal vasculature (*v*). **c**–**e** Confocal images of *tgfβ*-*f* expression in fertile adult colonies. Expression of *tgfβ*-*f* is present in follicle cells surrounding the maturing oocytes (**c**), primitive follicle cell-derived support cells around maturing testes (**d**), and circulating cells in the vasculature (**e**; *dotted lines* outline the vasculature). *Scale bars* indicate 50 µm. Nuclei are shown by DAPI staining (*blue*)

To better understand the factors and pathways regulating fertility in *B. schlosseri*, our laboratory compared the transcriptomes of fertile and infertile colonies and later expanded our analysis by comparing fertile and infertile colonies at each stage of asexual development [43]. By examining the genes upregulated in fertile animals, we took a candidate approach and performed in situ hybridization to identify genes associated with the germline or gonad development. One such transcript was predicted by ScanProsite [44] to contain a TGF-β domain, indicating that it is a ligand belonging to the TGF-β superfamily of genes. The TGF-β domain of this protein, hereafter referred to as Tgfβ-f, contains seven cysteines in conserved locations (Fig. 1a) involved in secondary structure and dimerization of other known TGF-β family members [45].

*Botryllus* anatomy is characterized by three asexual generations simultaneously developing and functioning in a colony at any given point in time (Fig. 1b). Furthermore, colonies contain numerous genetically identical individuals that are interconnected by an extracorporeal vasculature. Each adult individual in a colony, or zooid, is connected to one or more primary buds, which in turn give rise to secondary buds. In fertile animals, primary buds contain developing gonads, including oocytes and testes, as well as undifferentiated germ cells. Using in situ hybridization, we found that *tgfβ*-*f* was expressed by the follicle/support cells surrounding the developing oocytes and testes (Fig. 1c, d), as well as a small number of cells circulating in the blood vessels (Fig. 1e). We have previously reported this gene as a marker for these cells and as a positive control for in situ hybridization techniques in *Botryllus* [33].

**Fig. 2:**
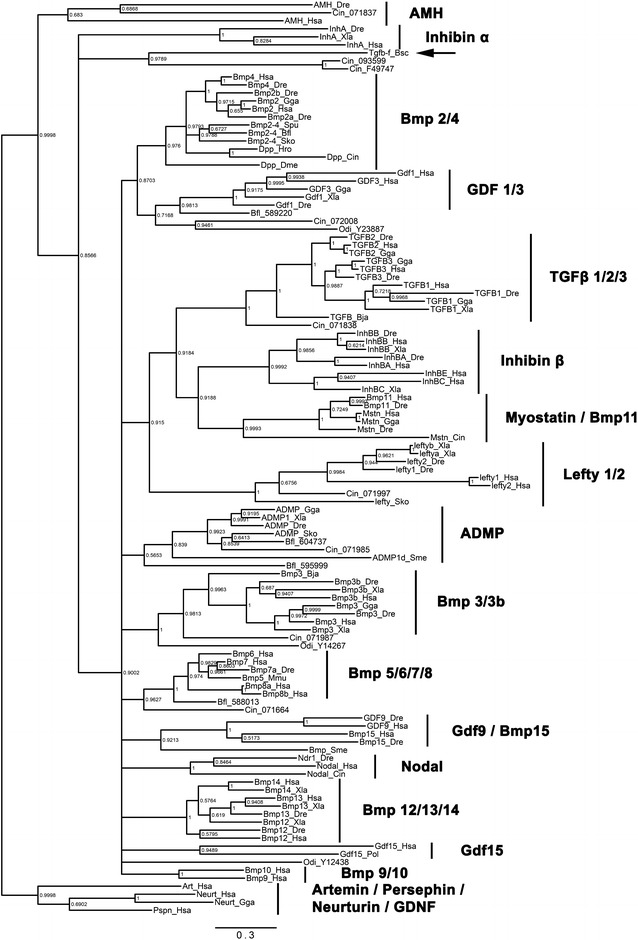
Bayesian phylogenetic analysis of Tgfβ-f and other TGF-β family members. Unrooted phylogenetic tree constructed by Bayesian analysis using MrBayes with four independent runs of one million generations, with trees sampled every 100 generations, 25 % of sampled values discarded as burn-in, and the mixed model mode. *Botryllus* Tgfβ-f is marked by an *arrow*. Families of TGF-β proteins are labeled and indicated by *vertical lines*. Nodes are labeled with posterior probabilities. The *scale bar* for branch lengths indicates expected substitutions per site

**Fig. 3:**
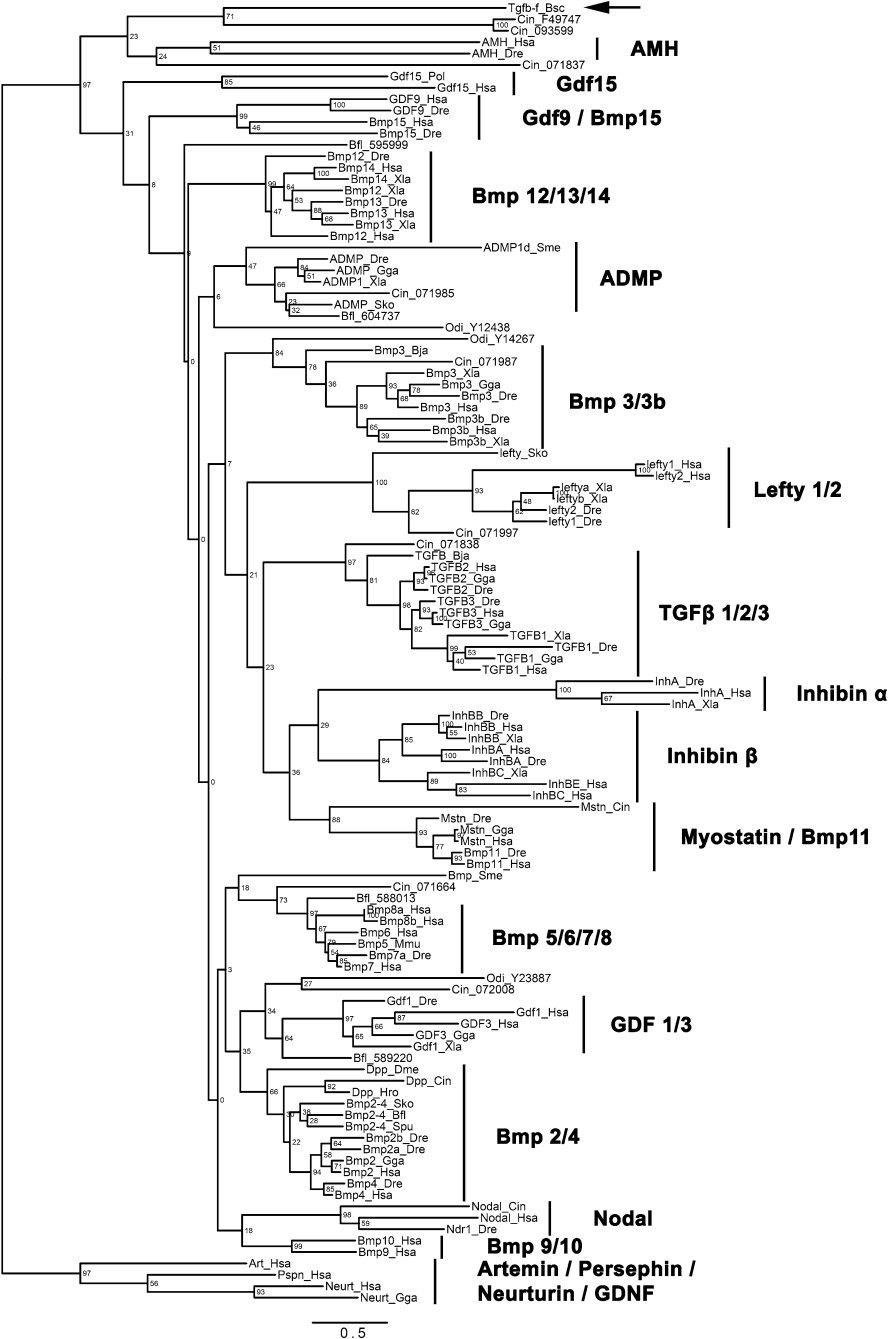
Maximum likelihood phylogenetic analysis of Tgfβ-f and other TGF-β family members. Unrooted phylogenetic tree constructed by maximum likelihood analysis using RAxML with 504 replicates for bootstrap, the JTT substitution matrix, and empirical frequencies. *Botryllus* Tgfβ-f is marked by an *arrow*. Families of TGF-β proteins are labeled and indicated by *vertical lines*. Nodes are labeled with bootstrap values in units of percentage. The *scale bar* for branch lengths indicates the mean number of inferred substitutions per site

In order to determine whether Tgfβ-f is related to one of the major clades of TGF-β family members, we performed Bayesian and maximum likelihood phylogenetic analyses. We aligned the full-length protein sequences of Tgfβ-f and 114 TGF-β family member homologs (85 vertebrate, 22 invertebrate chordate, and seven invertebrate non-chordate homologs) and removed gaps representing potentially poorly aligned regions using the software trimAl [37]. Our phylogenetic analysis robustly reconstructed the known subfamilies of TGF-β ligands, but intriguingly, we were unable to detect any support for Tgfβ-f belonging to one of these major clades (Figs. 2, 3) Instead, Tgfβ-f clustered with two unassigned *Ciona intestinalis* homologs, indicating that these proteins may represent a previously unidentified group of tunicate-specific or ascidian-specific TGF-β members. Interestingly, ESTs for one of the two *Ciona* proteins in this cluster, Cin_093599 (TGFbeta, not assigned 4; NCBI Reference # NP_001093599.1), have been detected in a cDNA library from the gonads of *Ciona* [46, 47], suggesting that the ancestor of Cin_093599 and Tgfβ-f may also have been expressed in reproductive structures.

### Tgfb-f-expressing follicle cell progenitors associate with germ cells in GFCs

**Fig. 4:**
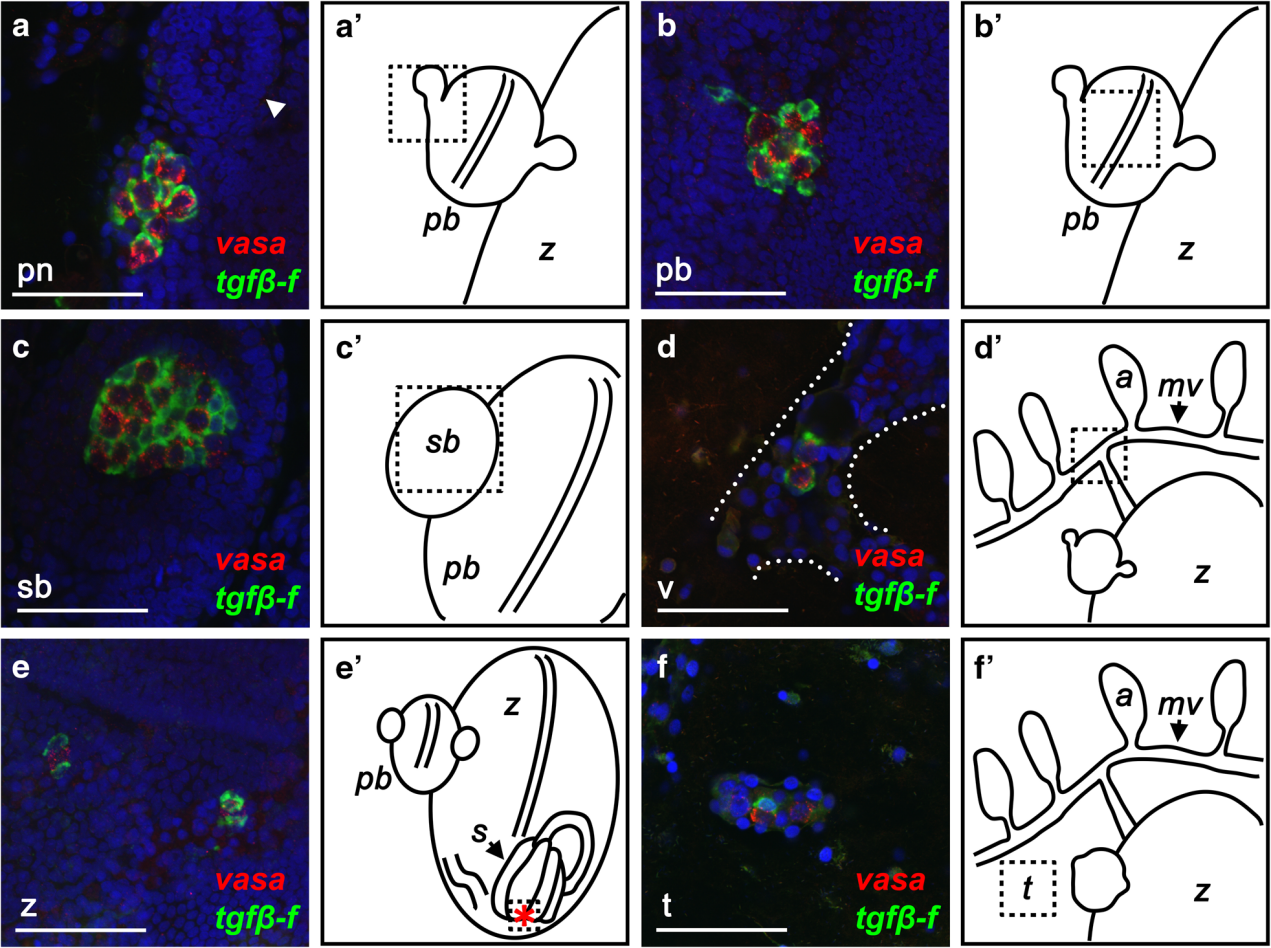
Locations where GFCs are found in *Botryllus* juveniles. **a**–**f** Confocal images of clusters composed of *vasa*-positive germ cells (*red*) and *tgfβ*-*f*-positive follicle progenitors (*green*) in *Botryllus* juveniles. **a** A cluster of germ cells and follicle progenitors in the primary bud niche (*pn*) is situated just posterior to a developing secondary bud at stage B2 (*arrowhead*). **b** A cluster of germ cells and follicle progenitors near the midline of a primary bud (*pb*) at stage B2. **c** A cluster of germ cells and follicle progenitors associated with the secondary bud (*sb*) at stage C1. **d** A cluster of germ cells and follicle progenitors in the vasculature (*v*; outlined by *dotted lines*) at stage B2. **e** Two clusters of germ cells and follicle progenitors near the stomach of an adult zooid (*z*) at stage C1. **f** A cluster of germ and follicle cells in a nodule embedded in the tunic of a stage A2 colony. *Scale bars* indicate 50 µm. Nuclei are shown by DAPI staining (*blue*). **a′**–**f′** Schematic diagrams indicating the general locations of each confocal image in **a**–**f** (*dashed boxes*). The *red asterisk* in **f′** marks the epithelium of the stomach’s posterior where GFCs are sometimes found. *mv* marginal vessel, *a* ampullae, *s* stomach

**Fig. 5:**
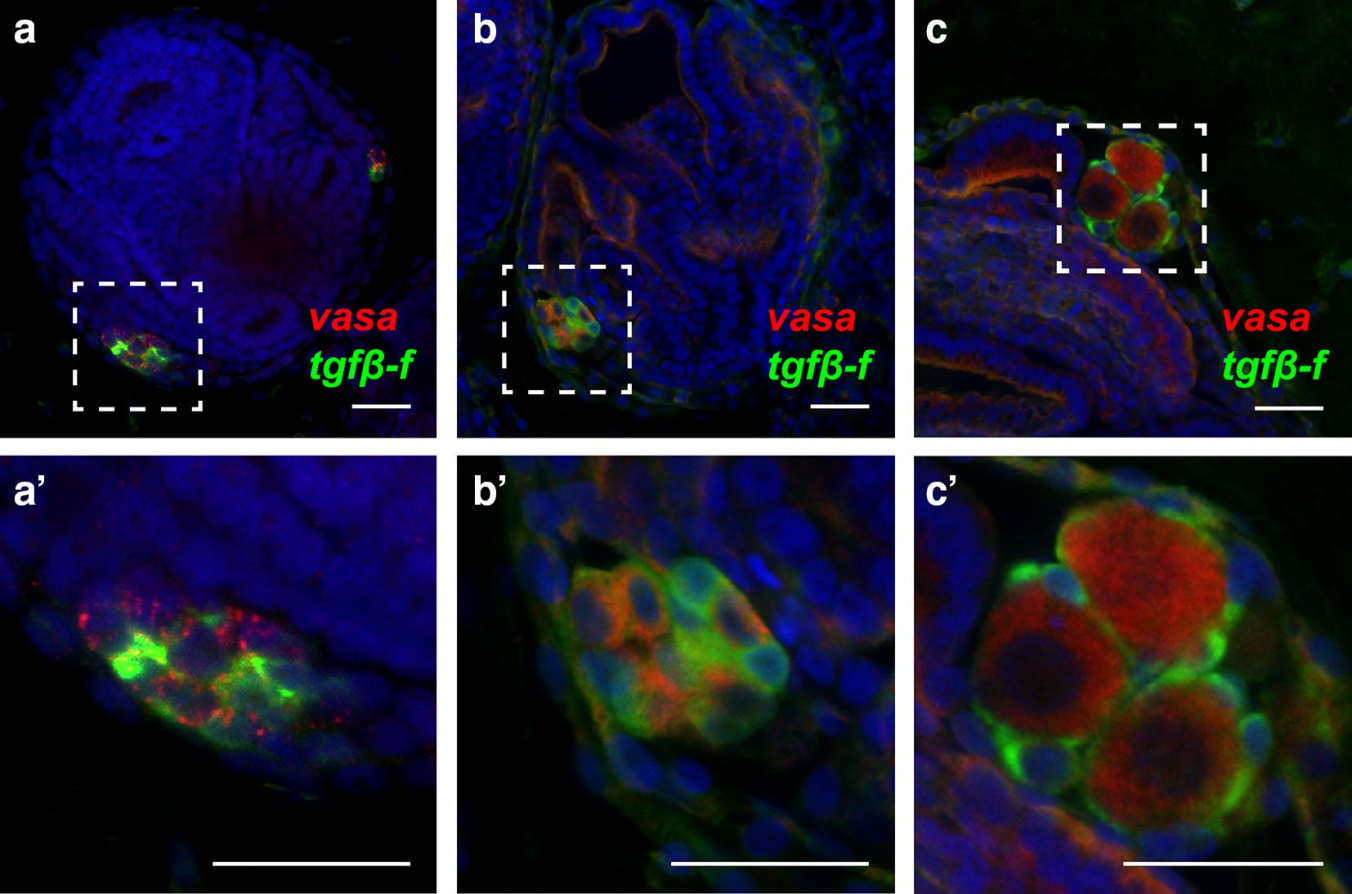
Organization of GFCs in oozooids, juveniles, and animals with maturing gonads. **a**–**c**, **a**′–**c**′) Confocal images of clusters composed of *vasa*-positive germ cells (*red*) and *tgfβ*-*f*-positive follicle progenitors (*green*) in *Botryllus* juveniles. The areas inside the *dashed lines* in **a**–**c** are displayed enlarged in **a**′–**c′**, respectively. **a**, **a**′ A GFC in a newly metamorphosed oozooid displays a lack of intracluster organization, with germ and follicle progenitor cells intermixed. **b**, **b′** A GFC in an infertile juvenile also shows intermixed cell types and no intracluster organization. **c**, **c**′ Follicle cells envelop newly maturing stage 2 oocytes in a juvenile. *Scale bars* indicate 25 µm. Nuclei are shown by DAPI staining (*blue*)

We next used double in situ hybridization to localize the expression of *tgfβ*-*f* with respect to *vasa*-positive germ cells in juvenile animals. We found that the majority of *tgfβ*-*f*-positive and *vasa*-positive cells were located in clusters in the primary and secondary buds of the colony (Fig. 4a–c, a′–c′). In newly metamorphosed oozooids and young juveniles, these clusters lacked organization and appeared to have *vasa*- and *tgfβ*-*f*-positive cells intermixed (Fig. 5a, a′, b, b′). As colonies matured however, *tgfβ*-*f*-positive cells were observed enveloping early-stage oocytes (Fig. 5c, c′). The location of the *tgfβ*-*f*-positive cells in juveniles and their association with *vasa*-positive germ cells indicates that they are follicle cell progenitors, which have been previously reported in botryllid ascidians [24, 48].

### Quantitative characterization of GFCs in juvenile Botryllus

In order to fully characterize the temporal and spatial localization of GFCs in *Botryllus*, we quantified these clusters and their constituting cells throughout the blastogenic cycle. Juvenile animals were used for our analysis because of their relatively small size and ease of staining and imaging, as well as the absence of differentiating gonads expressing *vasa* and *tgfβ*-*f* that could confound our identification of GFCs. From a total of 21 individual colonies containing 1–2 zooids, 1–3 primary buds, and 2–6 secondary buds/niches each, we analyzed 241 GFCs composed of 2682 *vasa*-positive germ cells and 2936 *tgfβ*-*f*-positive follicle progenitors. Interestingly, we found that the GFCs contained only these two cell types. Furthermore, nearly all germ cells were associated with follicle progenitors and vice versa (0.52 % of germ cells alone; 0.75 % of follicle progenitors alone), suggesting that GFCs in *Botryllus* can be formally defined as clusters consisting of *vasa*-positive germ cells and *tgfβ*-*f*-positive follicle progenitors.

We identified GFCs in six distinct anatomical locations in *Botryllus* colonies, including the primary bud niche, a site just posterior to the secondary bud where gonads develop in sexually mature animals (Fig. 4a, a′), associated with the primary bud itself (Fig. 4b, b′), associated with the secondary bud (Fig. 4c, c′), in the circulation (Fig. 4d, d′), associated with the adult zooid (Fig. 4e, e′), and within the tunic (Fig. 4f, f′).

**Fig. 6:**
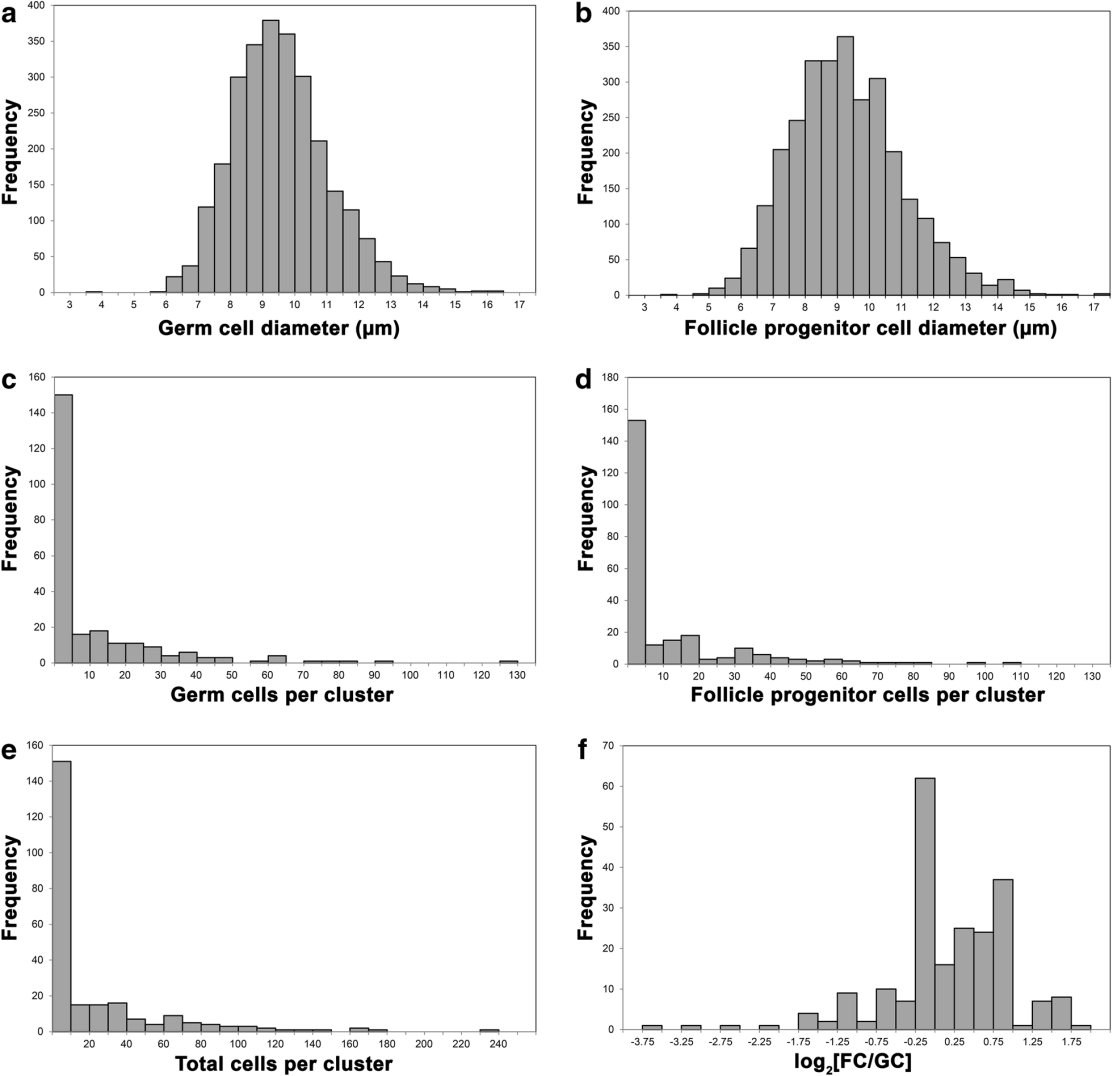
Quantitative parameters of germ cells, follicle progenitors, and GFCs. **a** *Histogram* of 2682 germ cell diameters from 21 total colonies, three from each stage of the blastogenic cycle. **b** *Histogram* of 2936 follicle progenitor cell diameters from 21 total colonies, three from each stage of the blastogenic cycle. Two outlying data points have been omitted from this *histogram* corresponding to diameters of 18.598 and 23.089 µm. **c** *Histogram* of the number of germ cells per cluster from 241 GFCs observed in 21 colonies at different stages of the blastogenic cycle. **d** *Histogram* of the number of follicle progenitor cells per cluster from 241 GFCs observed in 21 colonies at different stages of the blastogenic cycle. **e** *Histogram* of the total number of cells per cluster from 241 GFCs observed in 21 colonies at different stages of the blastogenic cycle. **f** *Histogram* of the log 2 of the ratio of the number of follicle progenitors (FC) to the number of germ cells (GC) in a given cluster, for 241 GFCs observed in 21 colonies at different stages of the blastogenic cycle

Using the extent of the mRNA expression of *vasa* and *tgfβ*-*f*, we estimated the diameter of each germ and follicle progenitor cell (Fig. 6a, b). Germ cells had a mean diameter of 9.56 µm with a standard deviation of 1.49 µm and ranged in size from a minimum diameter of 3.99 µm to a maximum of 16.24 µm. Follicle progenitors were similar in size to germ cells, with a mean diameter of 9.30 µm and a standard deviation of 1.75 µm. They ranged in size from a minimum diameter of 3.96 µm to a maximum of 23.09 µm. We also quantified the number of germ and follicle progenitor cells in each GFC. While the mean number of cells per GFC was 23.3, the majority of GFCs consisted of far fewer cells, with about 63 % of GFCs having ten or fewer cells (Fig. 6e). The numbers of germ and follicle progenitor cells per cluster were similarly skewed, with 62 and 63 % of GFCs having fewer than five germ or follicle progenitor cells, respectively (Fig. 6c, d). We compared the number of follicle progenitors to the number of germ cells in each GFC and found that this ratio exhibited a bimodal distribution, with peaks corresponding roughly to a 1:1 or 2:1 ratio of follicle progenitors to germ cells (Fig. 6f).

### Changes in GFC size and location accompany the change in location of the dominant germ cell niche

**Fig. 7:**
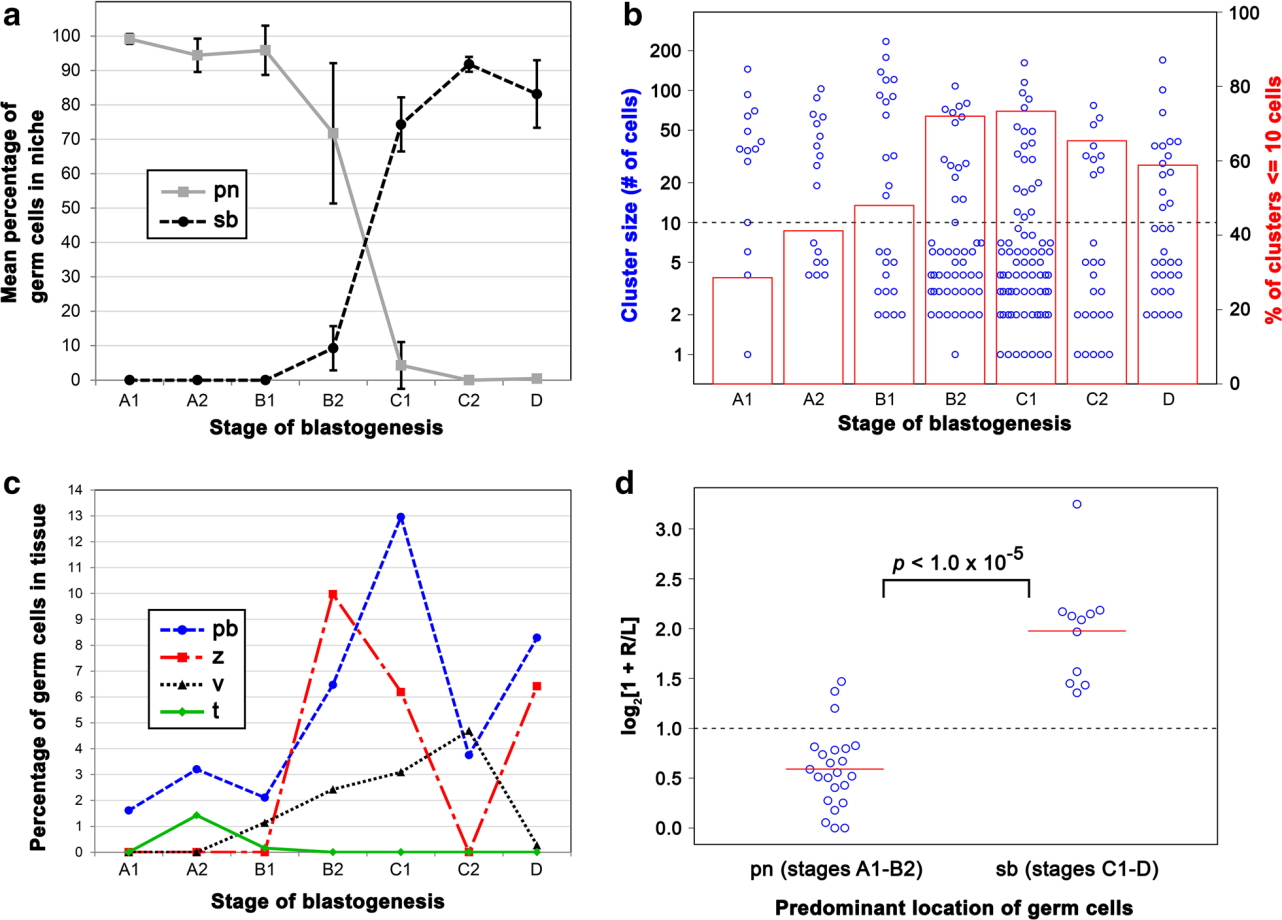
Dynamic sizes and locations of GFCs throughout the asexual life cycle. **a** Graph depicting the mean percentage (*y*-axis) of germ cells in the primary (*pn*; *gray solid* line) and secondary bud (*sb*; *black dashed line*) niches at each stage of the blastogenic cycle (*x*-axis) in juvenile *Botryllus*. *Each point* represents an average from three colonies, with *error bars* showing the standard deviation of these values. **b** Graph displaying GFC sizes (*blue open circles*, *left y*-axis with logarithmic scale) and the percentage of GFCs with ≤10 cells (*red bars*; *right y*-axis) at each stage of the blastogenic cycle in juvenile *Botryllus*. *Dots* representing GFCs with similar sizes are offset horizontally to prevent overplotting. The *horizontal dashed line* corresponds to a GFC size of ten cells. **c** Graph depicting the percentage of germ cells (*y*-axis) associated with the primary bud (*pb*; *blue long*-*dashed line*), zooid (*z*; *red two*-*dashed line*), vasculature (*v*; *black short*-*dashed line*), and tunic (*t*; *green solid line*) at each stage of the blastogenic cycle (*x*-axis) in juvenile *Botryllus*. **d** Graph depicting the log 2 of one plus the ratio of the number of germ cells on the *right* side of a primary bud to the number of germ cells on the *left* side of a primary bud (*y*-axis), for 34 individual primary buds (*blue open circles*) from stage A1 to B2 when most germ cells are localized in the primary bud niche (*pn*) and stages C1–D, when most germ cells are localized in the secondary bud niche (*sb*). One was added to each ratio prior to taking the log 2 since some primary buds had no germ cells on their *right* side. The means of the log 2 ratios were 0.59093 from stage A1 to B2 and 1.9761 from stage C1 to D and are represented by *red solid lines*. By Student’s *t* test, the means of these samples differed significantly, with a *p* value <1.0 × 10^−5^. The *dashed line* at *y* = 1.0 indicates the value at which an equal number of germ cells are present on the *right* and *left* sides, with values less than or greater than one indicating more germ cells on the *left* or *right*, respectively

**Fig. 8:**
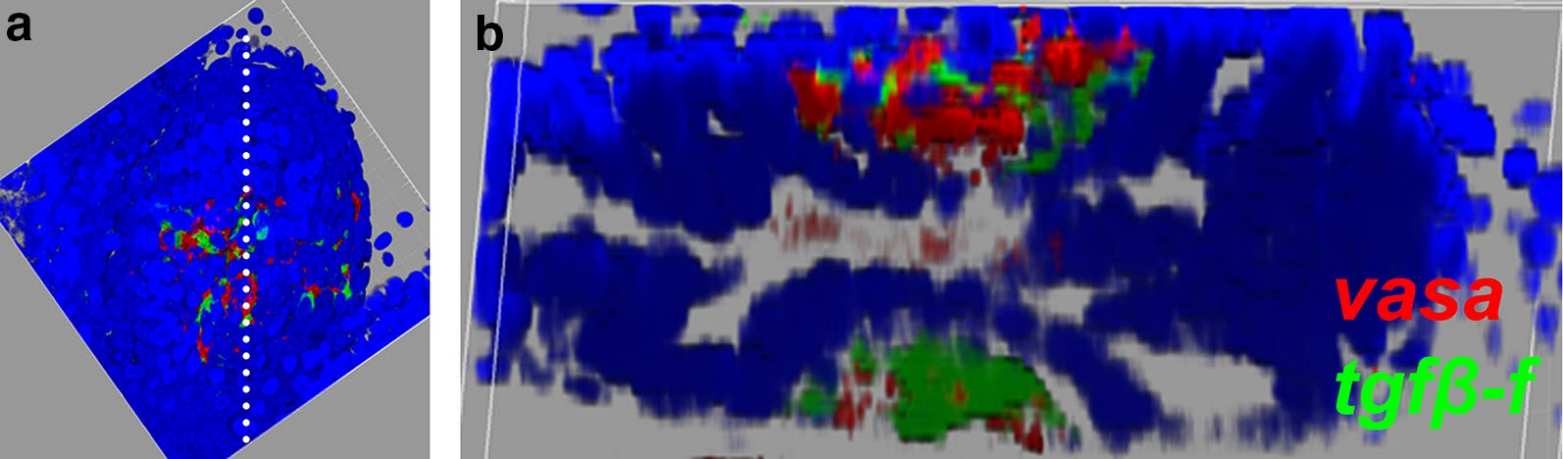
Germ and follicle progenitor cells form two main clusters in stage C1 secondary buds. **a** Ventral view of a three-dimensional reconstruction of a secondary bud containing *vasa*-positive germ cells (*red*) and *tgfβ*-*f*-positive follicle progenitors (*green*) at stage C1. Nuclei are visualized with DAPI (*blue*). **b** Optical sagittal section of the secondary bud shown in **a** along the *white dotted line*. Two GFCs are visible, one dorsal and one ventral

**Fig. 9:**
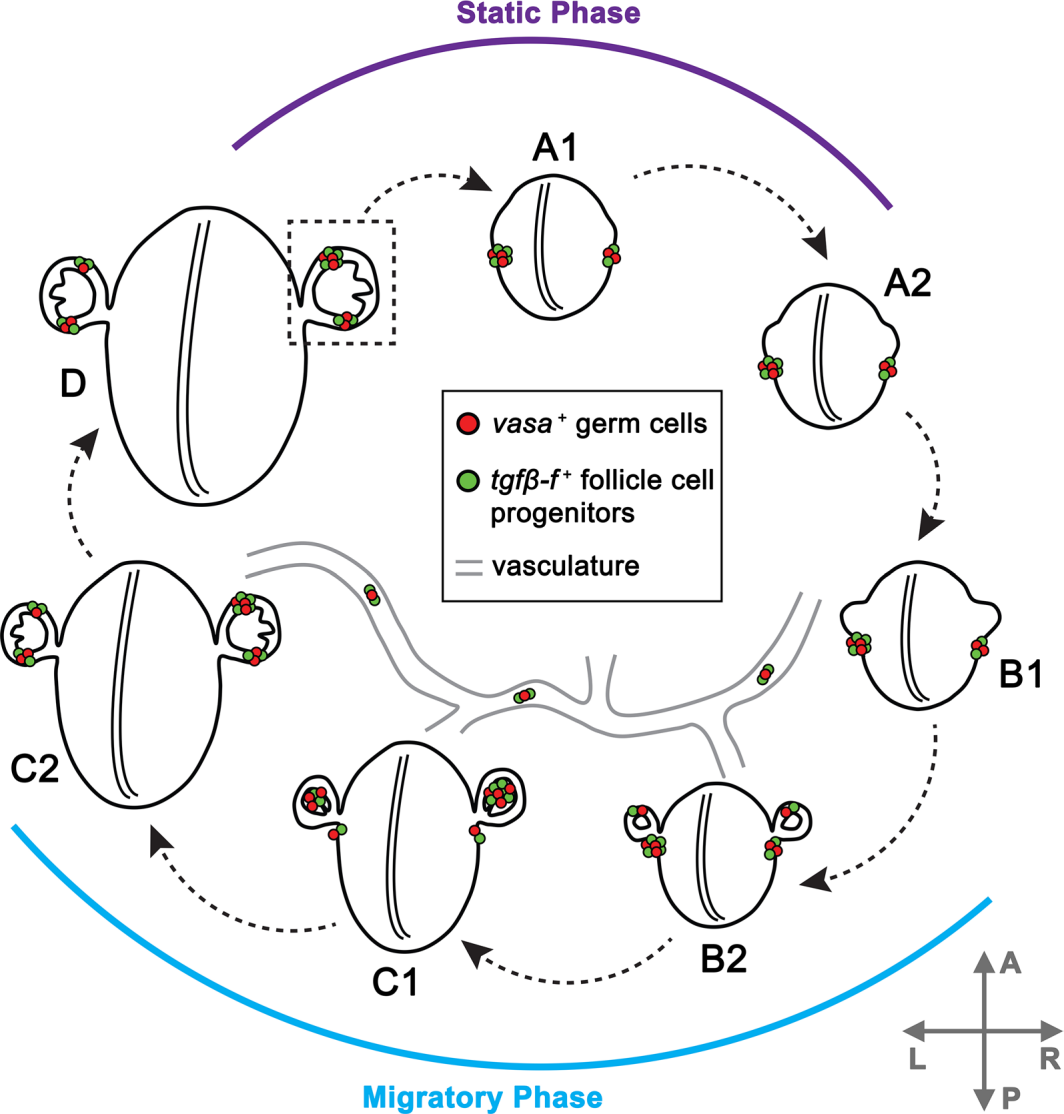
Schematic of GFCs during the asexual budding cycle. *B. schlosseri* has a 7-day asexual lifecycle called blastogenesis in which new bodies are formed by budding from existing ones. Three generations exist in a colony at any given time: functioning adult zooids, primary buds undergoing organogenesis, and newly formed secondary buds. This schematic shows germ cells (*red*) and follicle progenitors (*green*) in primary buds, secondary buds, and the vasculature (*gray*) at each of the seven stages: A1, A2, B1, B2, C1, C2, and D. During stages A1–A2, clusters of germ and follicle progenitor cells are almost exclusively found in the primary bud niche, just posterior to the developing secondary buds. At stage B1, GFCs are still predominantly found in the primary bud niches, but smaller GFCs can be detected in the vasculature. At stage B2, many smaller GFCs begin appearing, and GFCs can be found in the secondary buds and vasculature in addition to the primary bud niche. By stage C1, most germ and follicle progenitor cells are found in the secondary bud niches, and small GFCs can still be found in the vasculature. Two main GFCs are present in the secondary buds at this stage, one dorsal and one ventral. At stage C2, nearly all germ and follicle progenitors are found in the secondary bud niches, but some remain in the vasculature. Rotation of the developing secondary buds has oriented the two main GFCs of cells to anterior and posterior locations by this stage. During stage D, removal of the adult zooid occurs, and the secondary bud transitions into a primary bud. The two GFCs of germ and follicle progenitor cells in each secondary bud then seamlessly transition into the new primary bud niches of the next asexual cycle. *Axes* (*gray arrows*) show the orientation of the anteroposterior and *left*–*right* body axes. Germ cells exhibit *left*–*right* asymmetric abundance throughout the blastogenic cycle. *A* anterior, *P* posterior, *L* left, *R* right

Most germ cells (>75 % at any given stage of the blastogenic cycle) localized to one of two primary niches in the colony (Fig. 7a). During the early stages of the asexual lifecycle, before the secondary bud has formed a double vesicle (stages A1, A2, and B1), nearly all germ cells were restricted to the primary bud niche, a region at the periphery of the primary bud just posterior to the secondary bud (Figs. 1b, 7a). At stage B2, germ cells began to appear in the secondary bud (35 of 371 germ cells analyzed at stage B2) followed by a dramatic shift in the location of germ cells by the following day (379 of 517 germ cells in secondary bud at stage C1). Following this shift, the primary bud niche emptied of germ cells, and most germ cells were present in the secondary bud at stages C2 and D of the blastogenic cycle. The number of germ cells present varied substantially between secondary buds, with a minimum of zero germ cells and a maximum of 18 germ cells present per secondary bud at stage B2. From stage C1 to D, we observed a minimum of 11 germ cells and a maximum of 102 germ cells per secondary bud. However, despite this variability at the level of individual secondary buds, the fraction of germ cells present in the secondary buds of a given colony at each stage was more stable (Fig. 7a). In the transition from stage D to A1, the adult zooid regresses and is removed from the colony, the primary bud becomes the new adult, and the secondary bud becomes primary, generating new buds itself. Similarly, the germ cells associated with the secondary bud from stage C1 to D are located in the regions that will transition into the primary bud niches at stage A1. These GFCs are initially located dorsally and ventrally, but are reoriented by the subsequent rotation of the secondary bud (Figs. 8, 9). Thus, our analysis suggests that the change of the dominant germ cell niche is rapid, occurring in about 1 day, and that outside of this transition the localization of most germ cells is relatively static.

Accompanying the dramatic shift of germ cells from the primary bud niche to the secondary bud, we also detected an increase in the number of small GFCs (GFCs with ≤10 cells). Prior to stage B2, <50 % of the GFCs consisted of ten or fewer cells. At stages B2 and C1, a massive increase in the number of small GFCs was visible, with over 70 % of GFCs consisting of ten or fewer cells (Fig. 7b). We also noted an increase in the percentage of germ cells outside of the two major niches, and notably in the vasculature, at stage B2 and later. While the majority of vascular GFCs were present in the internal or marginal vessels of the colony, GFCs in the ampullae represented 29 % (8/28 GFCs) of all vasculature-mobilized GFCs during stages B1–C2. We also detected GFCs in the primary bud, adult zooid, and circulation, amounting to around 19 and 22 % of germ cells being outside of the primary and secondary bud niches at stages B2 and C1, respectively (Fig. 7c). These data suggest that around stages B1 and B2, GFCs may begin to fragment and enter a migratory phase that aids in the transition between the primary and secondary bud niches. Furthermore, the presence of GFCs in ampullae, the site of allorecognition in *Botryllus*, during stages B1, B2, C1, and C2 may indicate that transfer of the germline between fused colonies (germ cell parasitism) is favored during these stages. The difference in the percentage of small GFCs between stages D and A1 (59 vs. 29 %; Fig. 7b) also suggests that small GFCs may be removed as part of the takeover process, or that a period of rapid migration and coalescence occurs during this stage of the asexual cycle that was not observable by our whole-mount in situ-based analysis.

### GFCs exhibit a left–right niche preference prior to the onset of fertility

 Buds in *Botryllus* exhibit left–right asymmetry in organ situs and structure, as well as in their blastogenic and gonadogenic potential. In fertile adults, the left side of the primary bud has a greater gonadogenic potential, producing more eggs and testes, while the right side of the primary bud has a greater blastogenic potential, producing more successful secondary buds [11]. Intriguingly, we found that during the time frame when germ cells are predominantly localized in the primary bud niche (stages A1–B2), there is a strong bias for having a greater number of germ cells in the left niche versus the right (Fig. 7d). This left bias in juvenile animals mirrors the greater gonadogenic potential of the left side of the primary bud in fertile adults and opens up the possibility that this potential is the result of a greater number of germ cells being present to differentiate into gametes. Previous studies also noted an increased gonadogenic potential of buds originating from the right side versus the left [11]. We found that once germ cells were predominantly localized in the secondary bud (stages C1–D), there was a strong bias for having a greater number of germ cells in the right secondary bud versus the left (Fig. 7d). This right bias again mirrors the greater overall gonadogenic potential of right-derived buds and suggests that germ cell numbers may be responsible for the gonadogenic potential of a given niche.

## Discussion

### GFCs exhibit migratory and static phases

We have identified what appears to be a mobile niche for germline progenitors, a follicle progenitor cell type marked by expression of *tgfβ*-*f* that forms clusters exclusively with *vasa*-positive cells. Our analysis of these GFCs during the blastogenic cycle suggests that there are two distinct phases of GFC behavior during blastogenesis: migratory and static (Fig. 9). During stages A1–B1, most GFCs are found in the primary bud niches. However, at stage B1 GFCs also begin to appear outside of these niches and notably begin appearing in the vasculature. The vasculature in *Botryllus* is extracorporeal and connects bodies and buds throughout the colony. It has been previously suggested that the vasculature may be a conduit by which germ cells migrate to secondary buds from elsewhere in the colony [22, 29], and the timing of entry of GFCs into the vasculature, just prior to stage B2 when GFCs begin appearing in the secondary buds, supports this hypothesis.

Stage B2 is marked by a large increase in the number of small GFCs. We hypothesize that these smaller GFCs may be the result of fragmentation of larger clusters, since they form in the same time frame when the number and size of GFCs are decreasing rapidly in the primary bud niche and conversely increasing in the secondary bud (Figs. 7a, b, 9). Fragmentation of larger GFCs thus may be a key step in the migration of germ cells between niches in *Botryllus*. The functional importance of other locations where GFCs were detected, such as the primary bud and zooid, is unknown at this time, but it has been suggested that they may serve as transient microenvironments for migrating germ cells [31].

Similar to earlier work on germ cell migration in *Botryllus*, we observed GFCs associated with the stomach, intestine, branchial basket, and epithelium of adult zooids, as well as in regressing zooids at stage D [31]. However, in contrast to the findings in this earlier work that suggested migration of germ cells between niches occurs from stage D to A1, our data show that by stage C2, the secondary bud niche is more or less fully occupied, and further migration of germ cells is not needed to ensure that the next generation of buds has a germline. Germ cells coalesce in two locations (dorsally and ventrally) in the secondary bud by stage C1 (Fig. 8), and these two locations directly correspond to the future primary bud niches that will form after takeover, when the secondary bud becomes a primary bud and begins producing secondary buds of its own. Therefore, we hypothesize that a static phase in germ cell migratory behavior exists from stage D to A2, while an active migratory phase exists between stages B1 and C2.

While we did occasionally observe small GFCs nearby the endostyle, unlike previous studies we did not detect contribution of GFCs or *vasa*-positive cells to the blood cell islands (CIs), a putative niche for germ and somatic stem cells present in botryllids [31, 49]. However, several aspects of our analysis differed from these studies, potentially explaining the discrepancy. First, our analysis was carried out on juvenile *Botryllus* colonies instead of fertile adults. It is possible that GFC localization differs between fertile adults and juveniles. However, our analysis of *tgfβ*-*f* expression strongly argues against this possibility since we did not observe expression of *tgfβ*-*f* in fertile adult CIs. One notable difference between juveniles and fertile adults is that juveniles lack *vasa*-positive differentiating male and female gametes. It is conceivable that juveniles may also lack expression of *vasa* in a more stem-like germline progenitor that is present in CIs. Lastly, our in situ hybridization was performed whole mount on intact colonies while previous studies were performed on sections by in situ hybridization or immunohistochemistry [31, 49]. Differences in fixation and staining methods often affect the preservation of individual cell types and the signal detection in particular tissues and could explain the inability of our in situ hybridization method to detect *vasa*-positive stem cells or GFCs in the CIs.

While our data strongly suggest a migratory phase of GFC behavior, our analysis is limited by a lack of live imaging or lineage tracing. Instead of migration, a phase of germ and follicle progenitor cell specification may instead explain our findings. Epigenesis of germ cells has been detected during regeneration of tunicate species, including *B. primigenus* and *C. intestinalis* [24, 26, 50]. If epigenesis, rather than the migration of existing GFCs, is the primary mechanism by which new GFCs appear in the developing secondary buds, the appearance of GFCs in locations other than the primary bud niche and secondary bud may simply be an anomaly that is corrected by phagocytosis or cell death between stages D and A1. However, our finding that depletion of the primary bud niche and occupancy of the secondary bud occur in the same time frame suggests that at least some migration is likely happening between these locations. Alternatively, epigenesis and migration of existing GFCs may both occur during the asexual development of *B. schlosseri*.

### Relationship of Tgfβ-f to germ cell biology in Botryllus

TGF-β family ligands play important roles in reproductive biology in many organisms. In *Drosophila*, somatic cells surrounding germline stem cells in the ovary produce decapentaplegic (Dpp), a homolog of vertebrate bone morphogenetic proteins (BMPs). Dpp provides a crucial short-range signal that keeps germ cells undifferentiated, and dysregulation of this signal can result in the complete differentiation or uncontrolled expansion of the germ cell population [51–53]. In mammals, BMP15 and GDF9 are expressed by developing oocytes and regulate folliculogenesis and production of steroids by the surrounding granulosa cells [54–56]. Another key TGF-β family member involved in reproduction in vertebrates is anti-Müllerian hormone (AMH). In fish, loss of AMH signaling results in hyperproliferation of germ cells in both males and females [57]. In mammalian embryogenesis, AMH promotes apoptosis of the Müllerian ducts in males, preventing the formation of female reproductive structures. In adults, AMH is produced by Sertoli cells of the testes and ovarian follicular cells [58]. Activin and inhibin are complexes of TGF-β family ligands that play critical and opposing roles in fertility: Activin increases the binding and activity of follicle-stimulating hormone (FSH), thereby stimulating the maturation of germ cells, while inhibin inhibits the production of FSH [59, 60].

Our phylogenetic analyses did not suggest that Tgfβ-f is a member of any of the major families of TGF-β ligands found in metazoans (Figs. 2, 3), so we are unable to infer its function based on homology to a known protein. However, despite lacking clear orthologs, Tgfβ-f could have functional homologies to TGF-β proteins in other species. Given its expression in follicle progenitor cells, Tgfβ-f might function similarly to Drosophila Dpp or vertebrate AMH, regulating the differentiation or proliferation of neighboring germ cells. These follicle progenitors also accompany the germ cells during their apparent migration throughout the colony, and the signals they produce, like Tgfβ-f, may create a mobile niche-like environment where germ cell survival and proliferation are fostered. Expression of Tgfβ-f in support/follicle cells surrounding the developing testes and oocytes may also indicate a role in the maturation of the gametes. Antibodies directed against phosphorylated Smad2 and Smad1/5/8 proteins react with *Botryllus* germ cells and oocytes, indicating that they are receiving a TGF-β pathway signal [31, 61]. Future functional studies are needed to determine the role of TGF-β signaling and Tgfβ-f itself in *Botryllus* germ cell biology.

## Conclusions

Our findings indicate that the localization of GFCs is highly dynamic with respect to the asexual life cycle of *Botryllus* and suggest that these clusters may be migratory during stages B1–C2 of blastogenesis, and non-migratory otherwise. An increased number of small GFCs and the presence of GFCs in the vasculature coinciding with the appearance of GFCs in the secondary bud suggest that fragmentation of GFCs and the migration of smaller GFCs through the vasculature may be an important aspect of *Botryllus* reproductive biology, ensuring that the germline of one asexual generation is passed on to the next. Models like *Botryllus* are important for understanding the multitude of strategies that have evolved in organisms from different phyla to control germ cell specification, migration, and differentiation.

## Additional files

10.1186/s13227-016-0047-5 Species origins and GenBank or NCBI Reference IDs for protein sequences used in phylogenetic analyses. Column A contains the label given to the sequence in the phylogenetic trees. Column B contains the GenBank or NCBI Reference ID for the sequence used in the phylogenetic analysis. Column C contains the name of the species from which the protein sequence is derived.
10.1186/s13227-016-0047-5 Multiple sequence alignment in fasta format of Tgfβ-f and 114 other TGF-β family members from vertebrates and invertebrates. Protein sequences were aligned with MUSCLE and gaps were trimmed with trimAl. Genbank/NCBI Reference IDs for the aligned sequences, as well as the species of origin, may be found in Additional file 1: Data S1.

## Abbreviations

TGF-β: transforming growth factor beta
Tgfβ-f: transforming growth factor beta, family member f
GFC: germ/follicle cell cluster
DIG: digoxigenin
CI: cell island
Dpp: decapentaplegic
BMP: bone morphogenetic protein
AMH: anti-Müllerian hormone
FSH: follicle-stimulating hormone
